# Chemputation and the Standardization of Chemical Informatics

**DOI:** 10.1021/jacsau.1c00303

**Published:** 2021-08-31

**Authors:** Alexander J. S. Hammer, Artem I. Leonov, Nicola L. Bell, Leroy Cronin

**Affiliations:** School of Chemistry, University of Glasgow, University Avenue, Glasgow, G12 8QQ, United Kingdom

**Keywords:** Automated Synthesis, Chemical Informatics, Digital Chemistry, Data Standards, Reaction Optimization

## Abstract

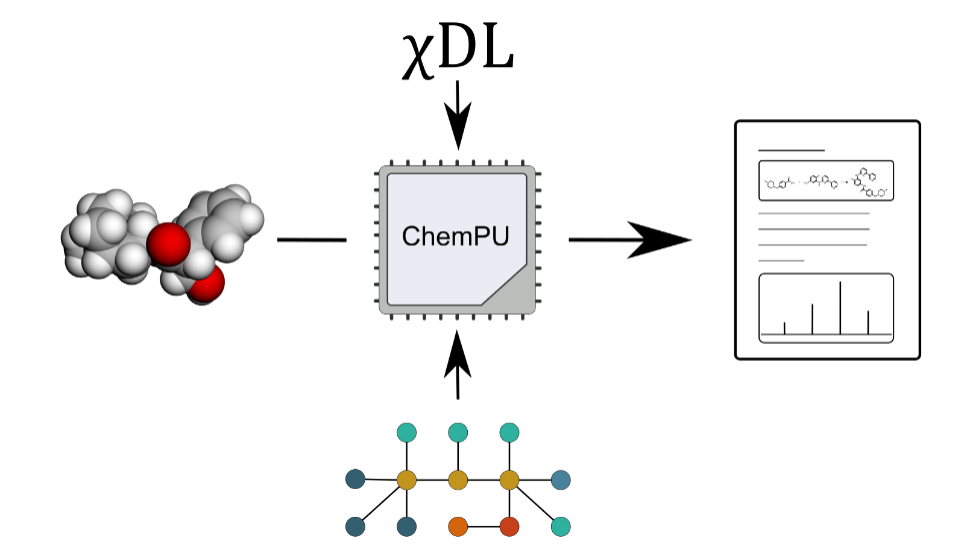

The explosion in the use of machine learning for automated chemical reaction optimization is gathering pace. However, the lack of a standard architecture that connects the concept of chemical transformations universally to software and hardware provides a barrier to using the results of these optimizations and could cause the loss of relevant data and prevent reactions from being reproducible or unexpected findings verifiable or explainable. In this Perspective, we describe how the development of the field of digital chemistry or chemputation, that is the universal code-enabled control of chemical reactions using a standard language and ontology, will remove these barriers allowing users to focus on the chemistry and plug in algorithms according to the problem space to be explored or unit function to be optimized. We describe a standard hardware (the chemical processing programming architecture—the ChemPU) to encompass all chemical synthesis, an approach which unifies all chemistry automation strategies, from solid-phase peptide synthesis, to HTE flow chemistry platforms, while at the same time establishing a publication standard so that researchers can exchange chemical code (χDL) to ensure reproducibility and interoperability. Not only can a vast range of different chemistries be plugged into the hardware, but the ever-expanding developments in software and algorithms can also be accommodated. These technologies, when combined will allow chemistry, or chemputation, to follow computation—that is the running of code across many different types of capable hardware to get the same result every time with a low error rate.

## 1 Introduction

**Figure 1:**
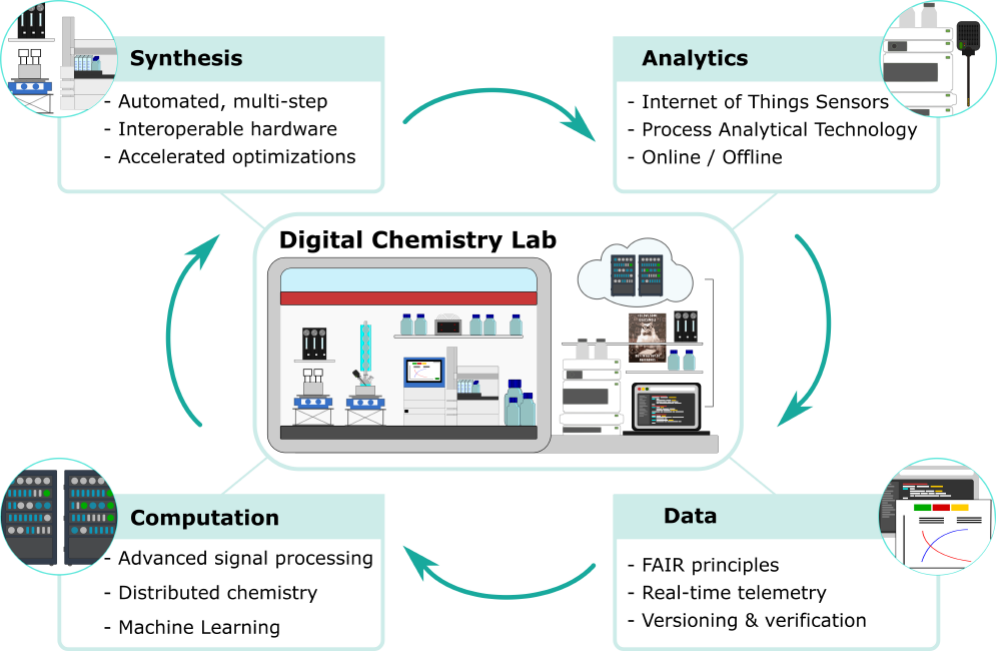
Digitization of a chemistry lab involves all aspects of lab work and data analysis. Automatic synthesis hardware should be able to undertake multiple unit operations or steps and use modular hardware that can communicate with different control systems and hardware systems. Analytics includes not only spectroscopy, spectrometry, and chromatography but also sensors for the continuous monitoring of process variables, such a temperature, which are vital input/output parameters for optimizations. Data must be collected, stored, and shared using FAIR principles such that both suboptimal and optimal conditions are recorded.

The exploration and optimization of chemical reactions can be a laborious and time-consuming endeavor^1^ as poor optimization strategies, combined with human intuition laced with biases, means that a large number of reactions must be undertaken to map a given chemical space.^2^ Worse yet, published and previously optimized methods have well-known reproducibility issues.^3^ A host of different procedures must be carried out in any chemical synthesis leaving significant room for miscommunication, lack of detail, or omission of tacit knowledge.^4^ In recent years, attention has turned to addressing these problems through initiatives which aim to normalize data generation and improve data sharing standards as well as applying novel methods for analyzing available reaction data, including machine learning.^5−11^ These efforts can be seen as first steps toward the full digitization of chemistry (Figure 1).^12^

**Figure 2:**
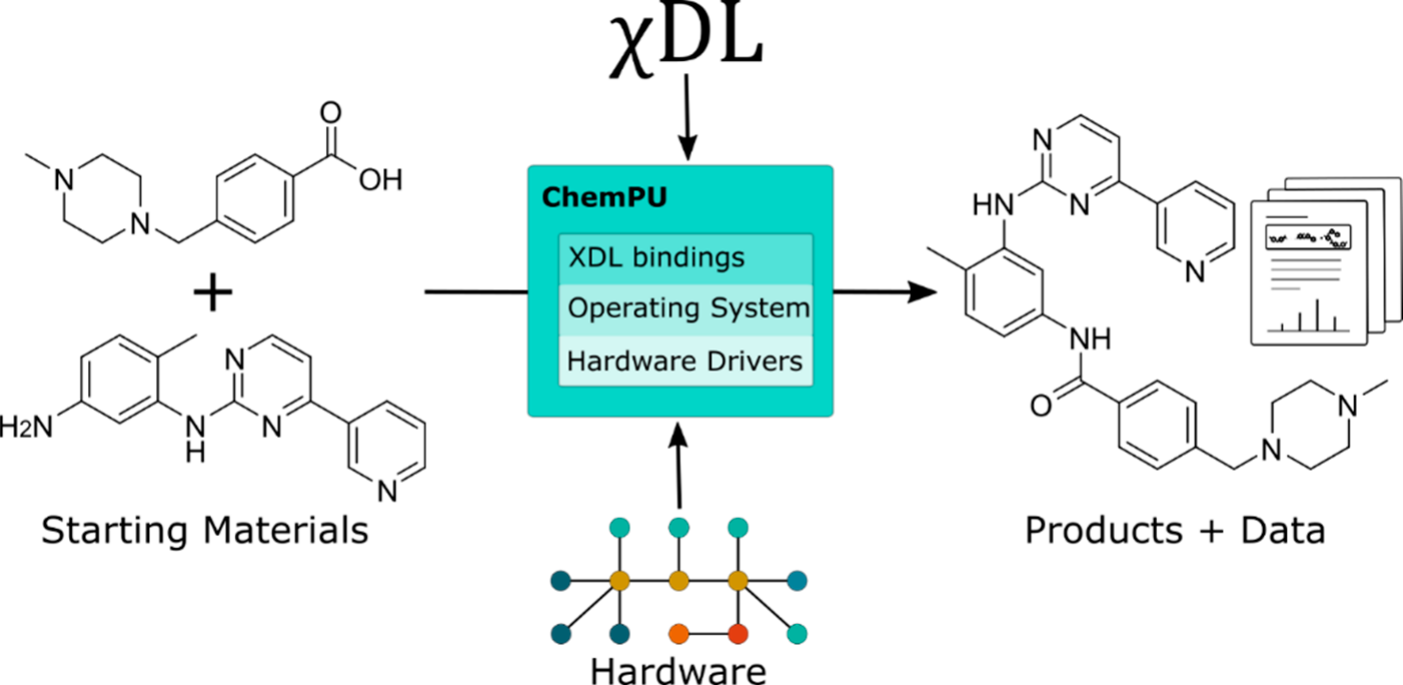
Chemputation: The process of converting a chemical code (χDL) to a reaction outcome with low error in any compatible robotic architecture, c.f. computation—running code on a digital computer. A chemical processing unit converts the inputs (starting materials, χDL code, and synthetic hardware) into outputs (reaction products, analytical data).

At first glance, the digitization of chemistry can be seen as the process of converting information, typically gained from physical experiments, into a digital format. However, on a deeper level the digitization of chemistry involves the full control of chemical processes by capturing all relevant input parameters, process operations and output data, and representing these in a machine-readable fashion to allow consistent reproduction of processes and efficient dissemination of the knowledge obtained.^13−16^ We envision that future digital chemistry laboratories will run automated, multistep reactions on a variety of interoperable hardware. Processes will be monitored with an array of sensors and process analytical tools, enabling rapid sharing of experimental data and advanced algorithmic control, which will be key to fully exploit new understanding and develop new models.^17,18^ In addition, for full exploitation of the potential of digital chemistry, a unified set of data formats, data capture standards and sharing guidelines are required to prevent research groups and fields becoming siloed. Incorporation of digital tools into the workflow of chemistry has already begun to increase efficiency, discovery, and the pace of innovation and it is clear that the volume of scientific data will rapidly proliferate in the coming years.^9,19−23^ In chemical synthesis and reaction analytics, it will be essential to capture all the key parameters of a preparation or experiment, including the context of the work. This approach will be essential for reproducibility, however, with only a few exceptions, this data has not been recorded reliably to date. Herein we describe how, in order to achieve this vision we need a standard architecture, comprised of a high-level machine and human readable code, to describe and record chemical process steps. It will be vital to connect this via a standard data structure to a wide range of affordable, modular synthetic, and analytical hardware (Figure 2).^4,14^ This architecture must also work in tandem with feedback algorithms to provide the key features required for process optimization and the acquisition of large reaction data sets.^24^

## 2 Digitizing Chemical Reactions

**Figure 3:**
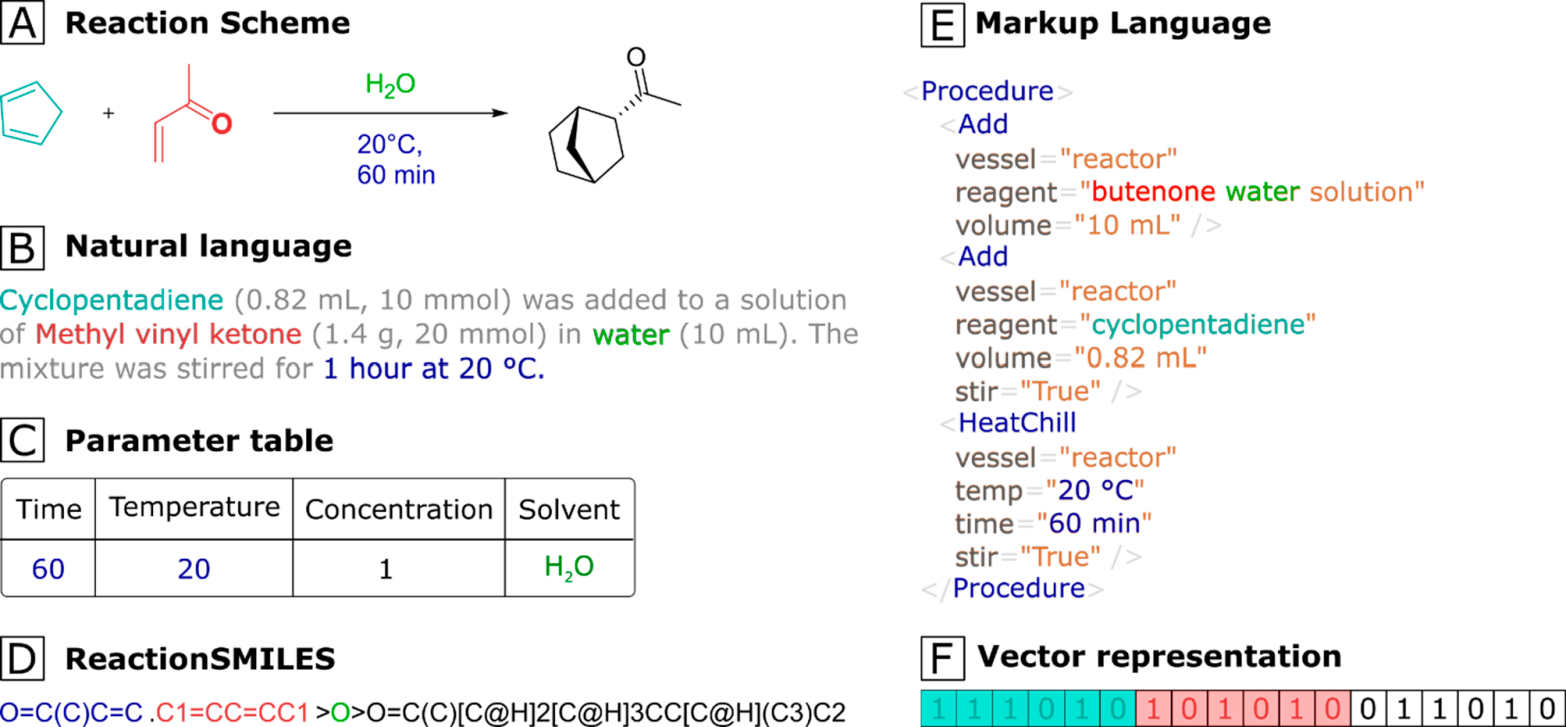
Different representations of the same reaction. Chemists commonly use Kekulé type reaction graphs (A) to depict reactions however these assume many procedural variables. More variables may be captured in prose (B), but this can be challenging for machines to interpret. Parameter tables are commonly used to capture optimization data (C). However, for digital chemistry representations, such as ReactionSMILES (D), vectors (F), or a markup language (E), which may or may not utilize the former, are favored.

To describe a reaction, chemists typically draw a reaction scheme made up of 2D graphs of the molecular structures comprising the reagents, any catalysts, and the products, see Figure 3A. Reaction conditions may or may not be indicated by inclusion of parameters in the scheme. While this may indicate key variables, much knowledge is assumed or omitted in these “schemes”. In addition, the actual description of how to perform the reaction in detail is often recorded in continuous prose in a different supplementary document to where the graph is documented, see Figure 3B.

In the context of reaction optimization, experiments are often carried out using a one factor at a time (OFAT) methodology and may be represented in a parameter table, listing the values of continuous (e.g., temperature, time) and categorical (e.g., solvent type) variables as well as the reaction outcome, see Figure 3C. Alternatively, design of experiments screening,^2^ spider plots,^25^ and traffic light systems^26^ have all been used to identify the specific parameters, which disproportionately influence reaction yield or any other optimizable metric. However, for all these methods, the decision regarding which parameters are varied or recorded during an optimization campaign is the choice of the chemist and is, therefore, somewhat subjective. In addition, an arbitrary threshold may be set for the optimal output value, and the process operation described by the variables recorded can also be ambiguous.

For cheminformatic applications, the simplified molecular-input line-entry system (SMILES) was developed as a simple single line notation representing the chemical structure of a molecule in ASCII strings.^27^ SMILES representations have now been widely adopted for digital representations of molecules and can be easily converted into graph-based representations of the structures as to enable easy interpretation by the chemist. In this context reactions are often represented as reaction SMILES where reactants are separated by a period (.) and the reaction arrow is indicated by “ ≫ ” with the option to insert “arrow conditions”, see Figure 3D. Notable alternative representations of reactions include RInChI,^28^ which extend the idea of the IUPAC International Chemical Identifier (InChI)^29^ toward reactions, reaction-data files (RDfiles), CSRML,^30^ Reaction-MQL (an extension of the molecular query language (MQL)),^31^ difference vectors based on molecular maps of atom-level properties (MOLMAPS),^32^ and condensed graph of reaction (CGR),^33^ which were developed as a pseudomolecular object that represents the reaction by indicating the bonds formed and broken.^34^

Most machine learning algorithms are designed to work with fixed-length vector representations such as concatenated structural fingerprints, a set of calculated descriptors, or learned representations, see Figure 3F.^35,21^ Graph-neural networks have been employed for reaction prediction tasks,^36^ but simple text representations in combination with advanced natural language processing (NLP) techniques have achieved a similar performance.^37^ String representations such as SELFIES or DeepSMILES have recently been developed to increase reliability and ease for data-heavy cheminformatics applications.^38^

A number of tools have been developed to translate the full synthetic protocols, written in natural language into a machine-readable format.^39^ Chemical tagger was developed as a rule-based text-mining tool and eventually led to the US patent and trademark office (USPTO) data set where the procedure is stored as an action sequence in the XML-based chemical markup language (CML), which was developed to allow both humans and machines to disseminate chemical data without information loss.^40−42^ In addition, data-driven approaches for converting text into action sequences have also been demonstrated.^43^ Despite this most procedures currently in the literature do not contain all of the information required to fully automate a procedure with factors such as stirring rate and the addition rate. Furthermore, other data, such as the actual temperature, pressure, humidity, etc., the reaction was conducted (rather than room temperature) could be critical for successful automation.

**Figure 4:**
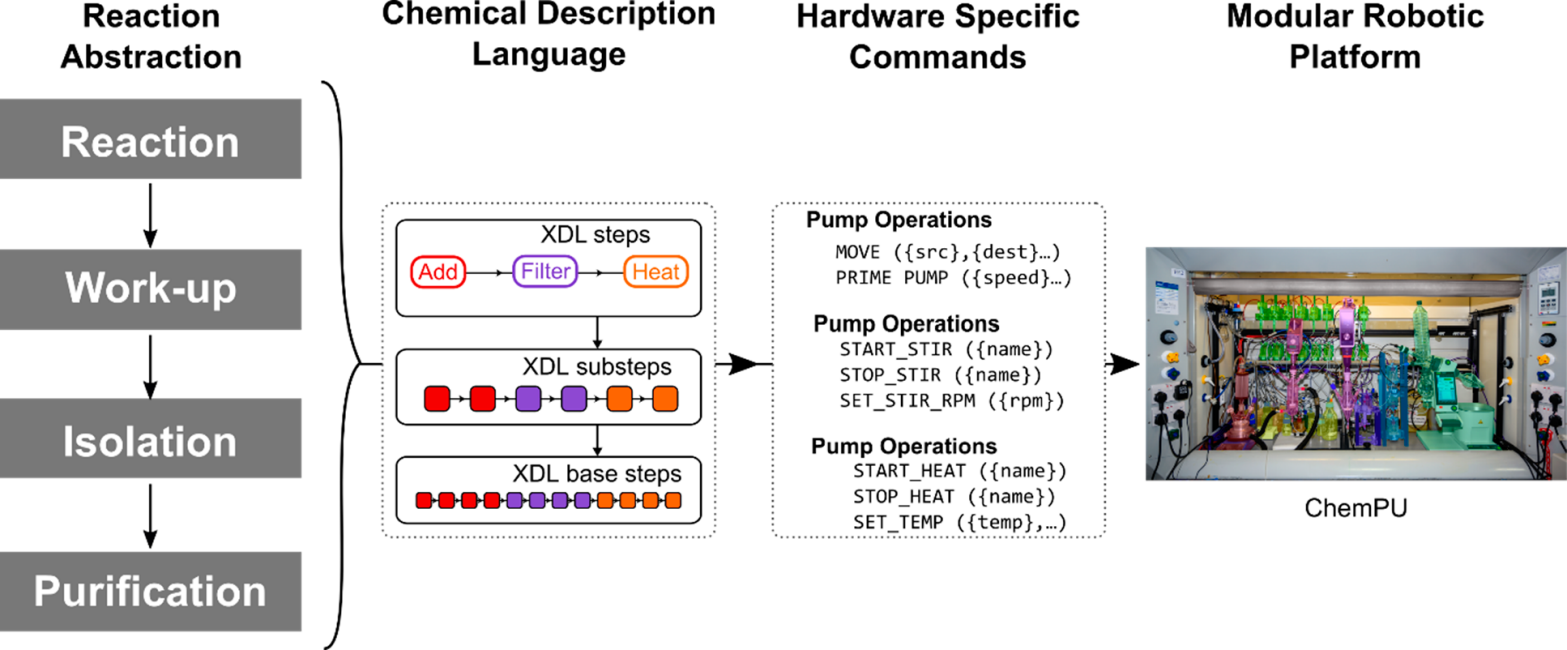
χDL files act as a bridge between the generalized abstraction of a chemical reaction process and the specific commands required for each platform or hardware.

In 2019, we introduced our invention of the Universal Chemical Description Language (χDL, see Figure 3E),^4,14,44^ in which all synthetic procedures can be encoded without ambiguity, executed on any compatible robotic platform, or manually, and exchanged using a standard format, see Figure 4.^4,45^ The aim was to expand on previous work by directly linking the sequences of actions to robotic execution on an automated synthesis machine.^14^ This was achieved by generating the universal abstraction of a batch chemical reaction. In general, all batch reactions follow the same procedure: (i) reaction, (ii) workup, (iii) isolation, and (iv) purification. Even complex stepwise synthetic procedures just follow the same abstraction in loops whereby one set of reagents is transformed into a set of products, and this is carried on to the next step and the process occurs again in a loop as the output products from step *n* become the reagents in step *n* + 1. This key recognition of the modular nature of the abstraction means it should be highly amenable to coding. The key is that many of the operations, once perfected, could be reused again. Furthermore, the abstraction being based on batch has parallels with computational architectures since the hardware could be set into specific states around the batch synthesis, whereas starting the abstraction in flow is harder. This is because flow processes are continuous which makes mathematical analysis difficult to make universal. However, the transformation from batch into flow is much easier because the frequency of the operations can be increased until they are continuous, and since these could have a discrete time stamp it will be easier to globally synchronize them. The instantiation of the abstraction requires the hardware modules to be capable of carrying out the operations described such that the hardware can be reset to the ready state (e.g., by cleaning) after each operation.

The modules that carry out the operations can then be represented on a graph as the nodes and these are given a network (IP) address, whereas the material transfer pathways (e.g., pipes moving liquids or path of a robot arm) are shown as the edges connecting the nodes together. The hardware set detailed in the graph has a defined set of unit operations they are each able to perform, while the χDL details all the unit operations required to undertake a given procedure. Thus, any robotic system with the required hardware modules organized appropriately on a graph can execute a given procedure by generating a platform specific executable file (XDLEXE) from the universal χDL. Importantly, χDL is simple and easily readable by both human and machine but can be produced automatically from natural language by extraction of action sequences from the procedures which allows the incorporation of key parameters such as order of addition.

Despite the vast wealth of chemical data contained in the literature, failed experiments are commonly omitted from the data published with articles in many journals^46^ even though this data has been shown to be valuable in predicting reaction outcomes.^47^ Most of this “failed” reaction data is currently stored, unindexed, in paper-based or electronic laboratory notebooks in academia or industry and is time-consuming to search even to those with access. With the advent of deep learning for chemistry, machine-readable data sets become increasingly important for computer-aided synthesis planning (CASP), automated synthesis and machine learning applications.^48^ Publicly available databases of chemical reactions remain rare with the most detailed and information rich databases, such as Reaxys, SciFinder, or Spresi, held behind paywalls (Table 1). For this reason, a consortium of academic groups and industry representatives was formed to develop the open reaction database (ORD) - an open-access repository of chemical reactions to fuel research in machine learning applied to chemistry.^49^ Despite this, the ORD does not intend to include digital action sequences geared toward automatic execution of reactions. In addition, analytical data (e.g., from LCMS, IR, NMR) is rarely available in an usable format from any of these databases. One vital component that is currently absent from the databases is the ability to store any run-time or current real-time data collected by sensors attached to the reaction hardware, for example, temperature probe on a stirrer hot plate to name a very simple example. We believe that the ability to collect real time data, or reaction telemetry, will be incredibly useful to both fingerprint successful reactions, record failure, and generate new insights and understanding. The so-called reaction-telemetry fingerprints that will be generated should have great potential to increase the robustness of automated workflows, validate robotically generated data for reaction optimization, and also aid in teaching and training of chemistry at all levels.

**Table 1:** Chemical Reaction Databases and Their Respective Data Classes Provided

	Reaxys	USPTO	Pistachio	Open Reaction Database	CAS Reactions
curator	Elsevier	D. Lowe/NextMove	NextMove	ORD Consortium	CAS
source	Gmelin, Beilstein, patents, papers	text-mined US patent grants and applications from 1976 to 2016	text-mined US and EPO Patents from all available years	public data sets + contributed data sets	curated from journals, patents, dissertations, etc.
size	>55 M	3.7 M	>9 M	NA	>136 M
failed reactions	N	N	N	Y	∼7 k
classification	Y	N	Y	Y	N
text	Y	Y	Y	Y	Y
conditions	Y	Y	Y	Y	Y
machine-readable actions	N	Y	Y	N	N
open access	N	Y	N	Y	N

Laboratory automation equipment is often expensive, highly specialized for certain tasks and only accessible to a small group of expert users.^50^ Open software and open hardware movements contribute to widening access but with an ever-increasing number of custom robotic platforms, cheminformatic workflows and file formats, there is an increasing need for standardization.^51^ The standardization in laboratory automation (SiLA) consortium was formed to develop principles that would enable plug-and-play laboratory automation.^52^ SiLA 2 was released in 2019 and is based on a microservice architecture and built to connect with laboratory information management systems (LIMS), electronic lab notebooks (ELNs), and other common laboratory software.^53^ For chemistry, ESCALATE (experiment specification, capture, and laboratory automation technology) was developed as a software pipeline to specify automated experiments and capture generated data in a structured manner.^54^ Research data should be findable, accessible, interoperable, and reproducible (i.e., FAIR) but since the inception of these principles by Mons et al. in 2016^55^ confusion on how to implement them has led to slow adoption.^56^ As HTE workflows become more commonly used to generate large amounts of data, there is an increasing need to validate and verify the data quality, for example, a simple hardware failure could lead to a large amounts of false negative reaction data.

In our experience, best practices for digital chemistry research include describing the experiment in a human- and machine-readable mark-up language which provides a high-level user interface, capturing exact versions of control software and dependencies, declaring the platform as a graph with all relevant metadata (such as type and volume of vessels, devices used etc.).^57,58^ The disconnect between process variables and actions, the suggestion of missing values and the removal of ambiguity in reaction data are currently bridged by expert chemists and need to be addressed by future standards. Importantly, the digitization of chemistry remains an ultimately human endeavor which is dependent on a change of the research culture. This means that university curricula must be adapted to provide education and training in digital technologies.^59^ Similarly, existing publication standards must be revised with new applications such as data mining in mind.

## 3 Automation of Chemistry

**Figure 5:**
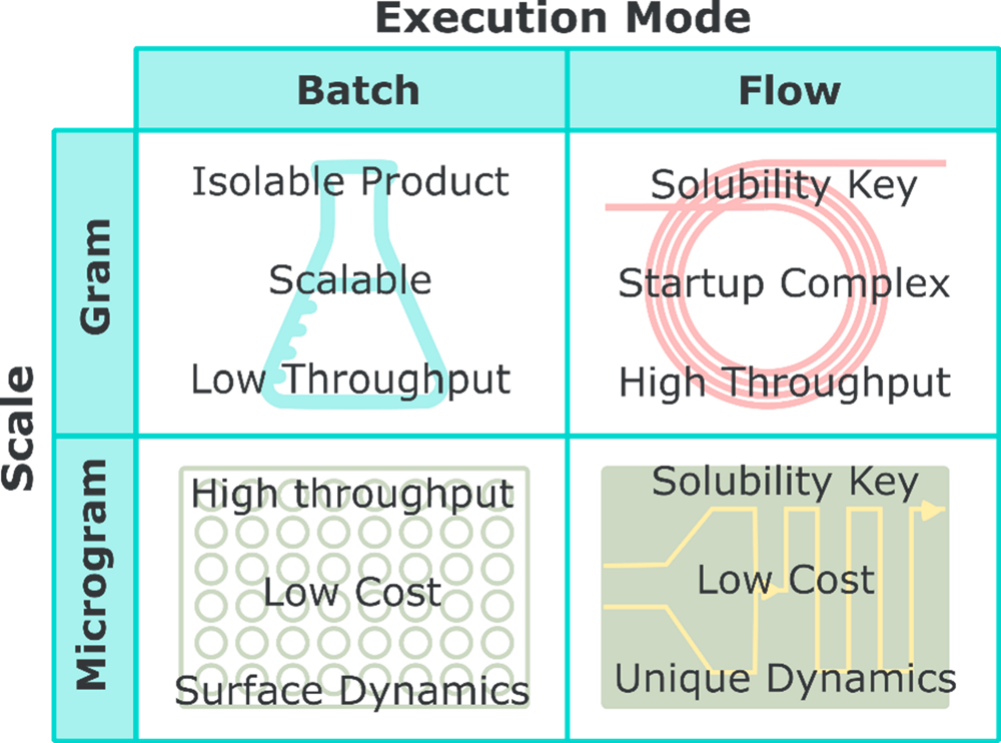
Automation hardware can be categorized by execution mode or by scale. Each has its advantages and disadvantages.

The synthesis of organic small molecules is still largely performed by hand in a laboratory setting that has barely changed in decades, but experts see the digitization of synthesis fast approaching.^13,60^ Merrifield pioneered the field by introducing the concept of automated solid phase peptide synthesis in 1965.^61^ The robust chemistry, simple purification procedure and iterative reaction cycles made the process amenable to automation. Smart laboratory automation holds promise to accelerate chemical research, eliminate tedious tasks, improve safety, and reliability.^62^ Indeed, the automated synthesis of oligopeptides, oligonucleotides,^63^ oligosaccharides,^64^ and metal oxides^22,65−67^ provided unprecedented access to these compound classes fueling exciting research in the areas of protein biochemistry,^68^ synthetic biology,^69^ chemical glycomics,^70^ and materials. Within the chemistry domain, automation may prove as a valuable tool for studying kinetics, optimization, discovery, and reaction telescoping.^10,71−73^ Over the last few decades, a range of synthetic hardware has become available to handle the throughput and procedural needs of autonomous optimization of chemical reactions (Figure 5).

Flow chemistry is one of the key enabling technologies for automation in chemistry.^74−76^ Ideally suited for solution-based reactions or those with immobilized catalysts, flow systems are easily automated as they rely on the simplest liquid handling robotics, i.e. syringe pumps. The ability to carry out multiple synthetic steps in tandem by facile addition of flow loops, to improve heat or light transfer and to append in-line analytics are all significant advantages for flow chemistry.^77^ These advantages were leveraged for the on-demand synthesis of multiple active pharmaceutical ingredients in a single robotic flow chemistry platform.^78^ While this approach required manually adjusting the platform to switch between different processes, a “radial synthesizer” was capable of performing multistep syntheses and optimizations for specific target molecules, as well as derivative libraries without instrument reconfiguration.^79^ The utility of automated flow for the optimization of cross-coupling reactions was demonstrated by performing over 5700 reactions on a micromolar scale in flow with inline HPLC analysis.^80^ Interestingly, it was possible to overcome the common inability of flow systems to meaningfully vary the reaction solvent by injecting a 9:1 ratio of diluent solvent to reagent stock solutions, achieving homogeneous mixing and screening the effect of solvent on the process. Jensen et al. elegantly demonstrated the advantages of flow automation for optimization with their “plug-and-play” modular flow reactor which features discrete loops for heating, cooling and photoirradiation.^7^ Despite these examples, current flow system architectures are not able to access full gamut of reactions available to batch chemists and parallelization of unit operations is required for continuous processing,^81,82^ meaning systems are only reproducible under equivalent flow conditions. In addition, in systems with metal-based flow loops, the role of the hardware in the chemistry may be noninnocent, adding significant complications for reproducibility.^83^ Finally startup and shutdown of these systems presents additional complications over beginning batch processes adding to the method development required for these syntheses. With the majority of reactions still performed in batch, significant method development is required to adapt known chemistry to flow platforms.

An alternative approach to automation is the combinatorial use of multiparallel batch reactors for high-throughput experimentation (HTE), which became popular in the early 1990s for the synthesis of large diverse screening libraries for drug discovery. This combinatorial chemistry and high-throughput screening paradigm was often blamed for a decline in productivity in the pharmaceutical industry,^84^ and yet it became a valuable research tool whose relevance is underlined by the commercial availability of mature XYZ Gantry (Chemspeed, Tecan) and ultralow-volume pipetting platforms (Mosquito). In recent years, we have observed a renaissance in the use of massively parallel, miniaturized ultrahigh-throughput experimentation^35,85−87^ combined with design of experiments (DoE) and other screening techniques applied to these reactors for discovering and optimizing novel reactivity, properties^88^ and even bioactivity,^89^ though not necessarily isolation procedures.^90^ These reactors, usually based on 96-, 384-, or 1536-well plate type designs, and allow hundreds of reactions to be run at once under the same process conditions.^80^ Despite this, the material consumption and waste were minimized because of the small reaction scale. While extremely powerful, this setup is far from general as there are significant experimental constraints imposed by the hardware (scale, compatible solvents, feasible temperature range) and such approaches appear to miss the flexibility required to automate multistep organic syntheses. Progress in recent years has led to significant progress toward a universal batch synthesis platform.^21,72^ The synthesis of many different types of small molecules in one automated process using *N*-methyliminodiacetic acid (MIDA) boronate building blocks could be accomplished by applying iterative synthesis similar to peptide synthesis enabled by a general MIDA catch-and-release purification protocol.^91^

**Figure 6:**
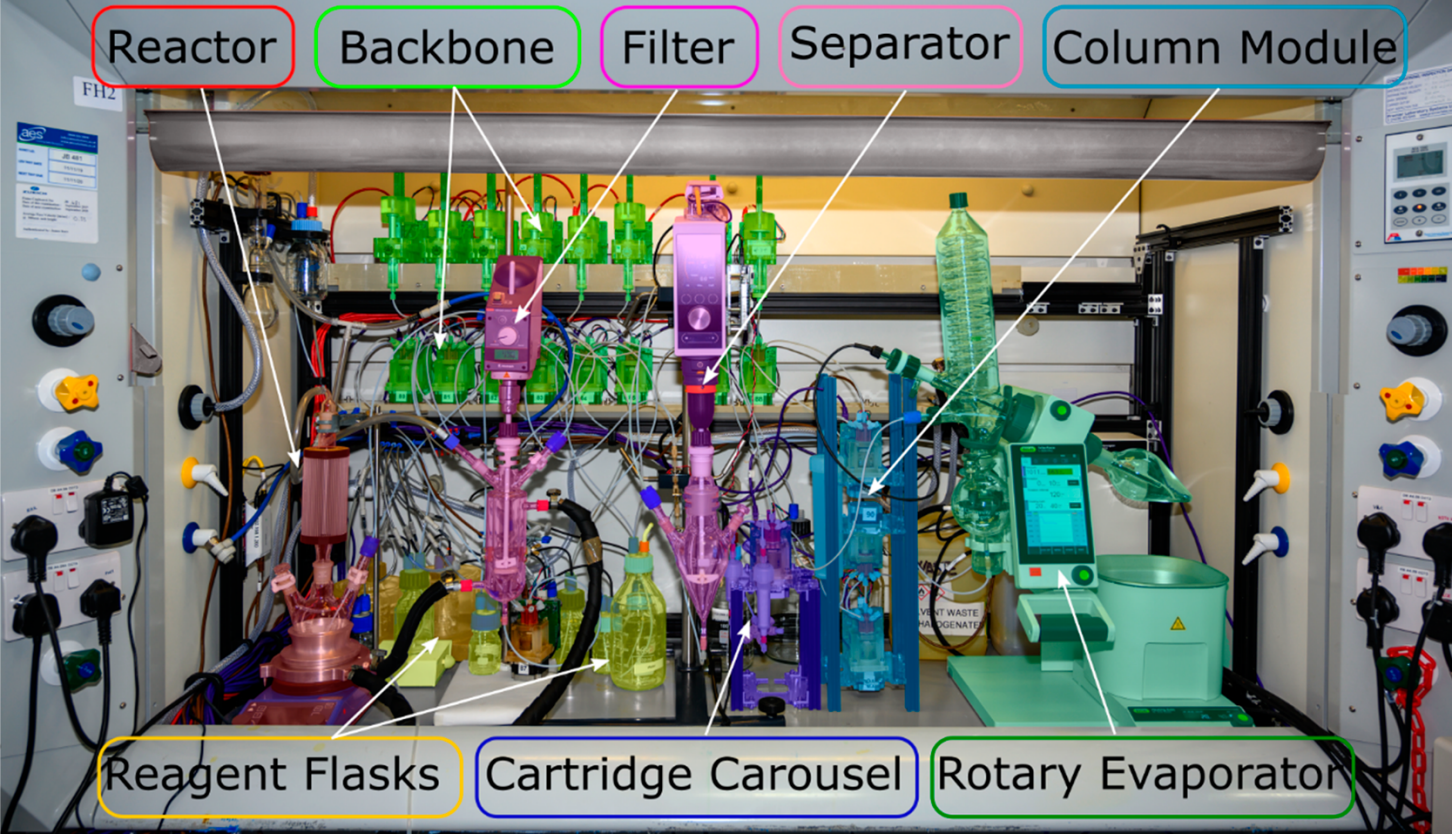
Photograph of the modular, universal ChemPU (Previously Chemputer) platform.

In 2019, we reported the development of a new approach to chemical synthesis architectures we first embodied in the “Chemputer” (now known as the ChemPU, Figure 6) providing standard software and hardware for complete automated synthesis and workup of a range of organic compounds based on a universal liquid handling backbone and modular additions for filtration, extraction, solvent evaporation, etc., see Figure 4.^14^ This platform emulates the traditional process operations, which would be carried out manually by a laboratory chemist and since the vast majority of the literature is based on batch chemistry, automation of these syntheses requires robotics founded in batch. Extension of the modularity of the original platform allowed for the execution of a wide range of chemistries on a single platform, including cross coupling, amide bond formation via peptide synthesis and diazirine formation.^57^ Importantly, the hardware modules, which make up each platform in the ChemPU family, are represented graphically and flexibly modified for each procedure using an online GUI. Both the hardware graph and the procedure are required for the generation of a platform specific executable to run the procedure. The procedure files for the ChemPU use the simple human and machine-readable chemical description language (χDL) which is hardware independent and therefore represents a universal chemical coding format. These χDL files describing synthetic procedures can be executed directly on the platform with minimal human intervention recording accurately every unit operation undertaken by the ChemPU.^4^ χDL files and hardware graphs can then be shared via Github upon publication allowing for others to reproduce these procedures exactly as performed in Glasgow even on different hardware as long as it meets the minimum standards for χDL compatibility.^4^

### 3.1 Control Systems

Simple experimental tasks can easily be automated using microcontrollers or single board computers with only a few lines of Python or Arduino (C++) code.^92^ While short linear scripts and hardcoded variables may suffice in some applications, robust and reusable software is needed to meet the reproducibility expectations of work that is to be classed as scientific research. Contrary to the expectation that digitization and automation should lead to increased reproducibility, code from many academic software projects does not execute, even just a few years after initial publication.^93^ This phenomenon is commonly known as “bit rot”, a term describing the apparent decay of software over time, can only be prevented by good software development practices and active maintenance by highly skilled programmers. This is hard to achieve in fast-paced research environments and with the high turnover of junior researchers in academia. Therefore, it cannot be expected that the end-user, most likely a chemist, is proficient in programming for broad adoption, but at the same time a well-designed user experience (UX) is of key importance.^94^ A simple standardized, flexible digital framework for chemical operations, interfacing with hardware, and controlled by a procedure code that links the operations to the framework is required for a digital revolution in the field because it could be designed with UX in mind, and in such a way that it could be maintained and developed collaboratively, c.f., Linux.

Commercial equipment often has proprietary software that comes with a user-friendly interface and, in some cases, basic scripting capabilities. However, closed platforms can lead to a vendor lock-in and impose barriers to innovation in research laboratories. For custom robotic workflows, open application programming interfaces (APIs) are therefore of great importance to integrate third party equipment and software.^95^ We believe independent bodies, such as the SiLA consortium, could and should establish standard APIs to interface with a wide variety of commercial laboratory equipment. LabVIEW by National Instruments (NI) is a generalized proprietary control software for a range of automation workflows integrating equipment from different third-party vendors.^96^ Its graphical programming approach allows users without programming experience to develop workflows via drag-and-drop. Furthermore, it is inherently concurrent, allowing for parallel execution and provides advanced signal processing capabilities. Since LabVIEW is limited in the areas of scientific programming including optimization, signal processing, statistics, and machine learning it is commonly coupled with Matlab.^7^ However, the ecosystem is considerably smaller than Python’s, for example, which is a general-purpose programming language and can be used to build sophisticated software solutions for automation-enabled or “self-driving” laboratories. Such solutions may combine chemical robotics with AI planning, database-management systems but also chatbots frameworks integrated in social media platforms for interaction with the human researchers.^22,97,98^

Hardware drivers, a platform operating system (OS), and bindings between this OS and the hardware-independent χDL form the software stack needed to run a ChemPU (Figure 2).^4^ This layered approach allows for simultaneous low-level access to the hardware for debugging and development purposes as well as high-level scripting capabilities for synthetic chemists with no programming experience using the χDL language. Importantly, this also allows the χDL to be run on different hardware for example using different drivers and OS, so long as equivalent bindings are present linking the unit operations detailed in the χDL to those performed by the hardware, see Figure 4. A web application, ChemIDE, was also developed as a human-friendly graphical user interface to allow chemists to directly develop chemical programs with little or no programming experience.

## 4 Data Collection

**Figure 7:**
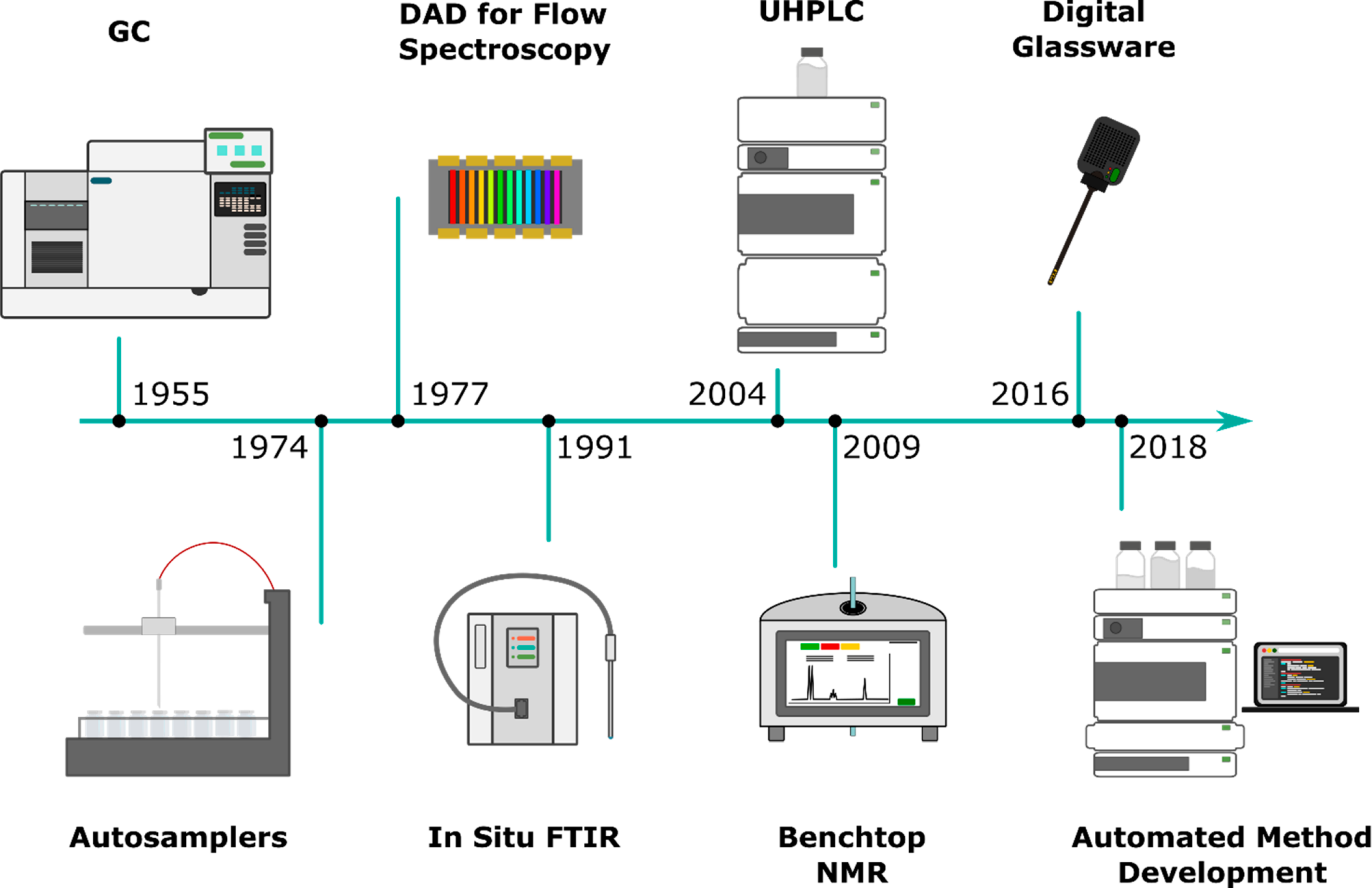
Advances in enabling analytics for automated reaction optimization. The earliest automation system was devised for gas chromatography (GC) with automatic flow rate and temperature control however automation really drove advances in science with the advent of autosamplers which allowed for hundreds of samples to be analyzed with minimum labor, freeing up scientists for data analysis. The ability to conduct analysis in flow has also been a strong driver of automation with diode array detectors (DAD) for UV–vis being among the first advances to facilitate this. In situ analytics, such as Fourier transform infrared spectroscopy (FTIR) and Digital Glassware, also allow for rapid or continuous monitoring of reactions in real time while UHPLC and benchtop NMR both cut the time and effort required for more advanced analytics. Currently method development for chromatographic separations is still a key bottleneck but new automated method development systems are becoming available to optimize separations autonomously.

Rapid real-time analytics are fundamental to optimization and a range of techniques have been developed to facilitate this alongside hardware advances which allow their incorporation into automated systems (Figure 7).^99^ Perhaps even more so than for synthesis, standardization of analytical hardware and data formats is vital and improving data standards means, increasingly, analytical data is available alongside publications, however, the facile machine readability of this data and interpretation in context of its source hardware still present challenges in developing standards. Inline Raman and IR spectrometers, in particular React-IR, have significantly increased the impact of this low energy spectroscopic method for real-time reaction monitoring and thus rapid feedback for optimization.^100−102^ The short time scale of the IR experiment means a vast number of data points can be collected for each reaction and the ability to home-in on particular functional groups of interest, to the absence of noise created by other reagents, makes this a useful technique for use in autonomous digital optimization. UV–vis methods also have the advantage of allowing one to focus on a specific wavelength; however, the resolution of UV–vis versus for example, IR makes analysis of this data more challenging. These methods may thus find more use in the optimization of inorganic compounds where a small difference in coordination environment leads to significant changes in the UV–vis spectrum.^103^

However, analyzing the shape of a time-dependent UV–vis absorption plot at a fixed wavelength has been shown to be useful for peptide synthesis.^104^ The inclusion of the time dimension allows a range of additional parameters be elucidated including reaction rate and reagent diffusion with different deprotection reagents and thus optimized using deep learning data analysis approaches. Gas and high-pressure liquid chromatograph methods are both well suited for automation, in-line analytics and subsequent reaction optimization and can be easily paired with other analytic techniques (e.g., UV–vis, MS).^7^ However, several key factors limit their use. Both methods require long experiment times, preventing high-throughput analysis, and they may require method development to ensure valid data is achieved when varying reaction parameters. In addition proprietary software and equipment means that the experimental methods and hardware modules are significantly less standardized across different systems than for IR and UV–vis spectroscopies.^105^

Finally, the information content of chromatographic methods alone can be low, particularly if they are not utilized in combination with analytical standards. Ultrahigh pressure (UHPLC) and techniques, such as flow injection analysis (FIA-MS),^106^ multiple injections in a single experiment (MISER),^105^ and 2D-LC analysis, have been developed to reduce experiment times for HTE.^107^ Agilent have also recently begun producing an automethod development system, InfinityLab, incorporating up to 8 different columns and 15 mobile phases for rapid autonomous chromatographic method development.^108^

Mass spectrometry for optimization is most often coupled with a chromatographic method allowing for more accurate quantification of relative yields and clearer identification of byproducts. For MS alone the high sensitivity and low sample mass requirements are a significant advantage, however, long experiment times can also be a factor here. Mass spectrometry has also been applied in flow systems for optimization of reactions, which allows the incorporation of feedback loops in the synthesis and, thus, more autonomy in the optimization process.^109^ Benchtop APCI MS has been utilized to optimize the formation of nicotinamide in such a system, using only 18 experiments, with yield calculated by normalization of the [M + H]^+^ adducts.^110^

Traditional NMR spectroscopy is based on batch analysis, with autosamplers allowing for more rapid throughput of samples; however, the recent inception of flow-NMR has truly allowed the technique to proliferate for automated reaction optimization.^111^ Flow NMR has the advantage of having highly transferable data analysis, high information content, and moderate acquisition times (∼0.5 s scan^–1^).^112^ The variety of nuclei, techniques, and ongoing advances also makes this method widely applicable to different chemistries, which may not be suitable for MS or chromatography.^113^ For example, insights gained from mechanistic analysis can inform reaction optimization and flow NMR has been utilized for the in situ study of reactive organometallics representing catalytic intermediates.^114^ Even common challenges in NMR such as overlapping peaks have been overcome utilizing state of the art algorithms.^115^

A prerequisite for autonomous operation of any laboratory equipment is dynamic feedback. While condition monitoring and process control are routine tasks in chemical engineering, it is much less common in academic chemistry research laboratories.^73^ Tasks such as measuring the pH value or ensuring a flask is empty are trivial for a human researcher but challenging and crucial for the safe operation of automated laboratory equipment. Data from “human monitoring operations”, such as the visual inspection of a reaction (e.g., to determine if precipitation or a color change has occurred), are not consistently captured. Emerging technologies enable chemists to acquire, share, and analyze digital data sets of their chemical experiments (Table 2).^92,116,117^ Recent examples of this development are the “Smart Stirrer” capable of measuring reaction conditions, such as temperature, conductivity, visible spectrum, opaqueness, stirring rate, and viscosity in situ, and the use of low-cost optical bubble sensors to control a hydrogenation reaction. Also, the open-source software package Heinsight, which uses webcams and computer vision algorithms to monitor liquid levels, is being used for diverse applications, such as continuous preferential crystallization, slurry filtration, and solvent swap distillation.^118−120^

**Table 2:** Sensors Which Can Contribute to Automation of Standard Laboratory Procedures

sensor	example use case
pH	“Adjust the pH to 7.0 with NaOH (1 M).”
conductivity	“The solution was extracted with ethyl acetate.”
bubble	“Check if line is clogged.”
viscosity	“Heat until gelation occurs.”
turbidity	“Add hexane until a fine precipitate forms.”
liquid level	“Notify if waste receptacle is full.”
temperature	“Add dropwise, maintaining the temperature below 0 °C.”
color	“Stir until the color changes from red to blue.”

One important point which arises for all of these analytical systems is the need for robust data sharing processes upon publication.^121^ X-ray crystallography represents a case study in best practice with regards to analytical data sharing. Currently, an article containing an X-ray diffraction crystal structure must include a crystallographic information file (cif) incorporating all of the details of the equipment used, experimental parameters, raw data and processed output.^122^ All crystallography software is unified in its ability to produce identical output files and read identical input files and this data is stored in the easily visualizable and searchable Cambridge Structural Database as well as alongside the corresponding publications.^123^ At publication this file is subject to CheckCIF standards checks, a report of which must be provided to reviewers and editors.^124^ For other analytical data types, no such requirement exists but universal data standards for other methods, the quality of which can be validated, would be beneficial to the whole community. FIDs from NMR spectrometers,^125^ CSV files from spectrophotometers, mzML files from mass spectrometers,^126^ and similar data from GC/HPLC systems should all be a minimum requirement for publication of results, which rely upon this data.

Only through the peer review system can we effectively ensure data standards are maintained across the discipline. One feasible example format for sharing data at publication could be the JCAMP-DX format which has been demonstrated for use in all these analytical techniques and more, with these files detailing both spectral parameters and metadata.^127^ An alternative format could be the Analytical Information Markup Language (AnIML)—a XML-based solution for storing analytical data from a variety of instruments and techniques, offering a validation process via strictly defined schema.^128^ Our vision includes the sharing of analytical (e.g., in aforementioned format) and process (in tabular format, e.g., a csv file, with the timestamps for each measurement) data alongside the χDL process file. Thereby each “version” of data created by a new experiment run includes all data for that run, including any failed runs, and specifications/parameters on all the hardware used to collect such data alongside the procedural information contained in the χDL.

## 5 Optimization

**Figure 8:**
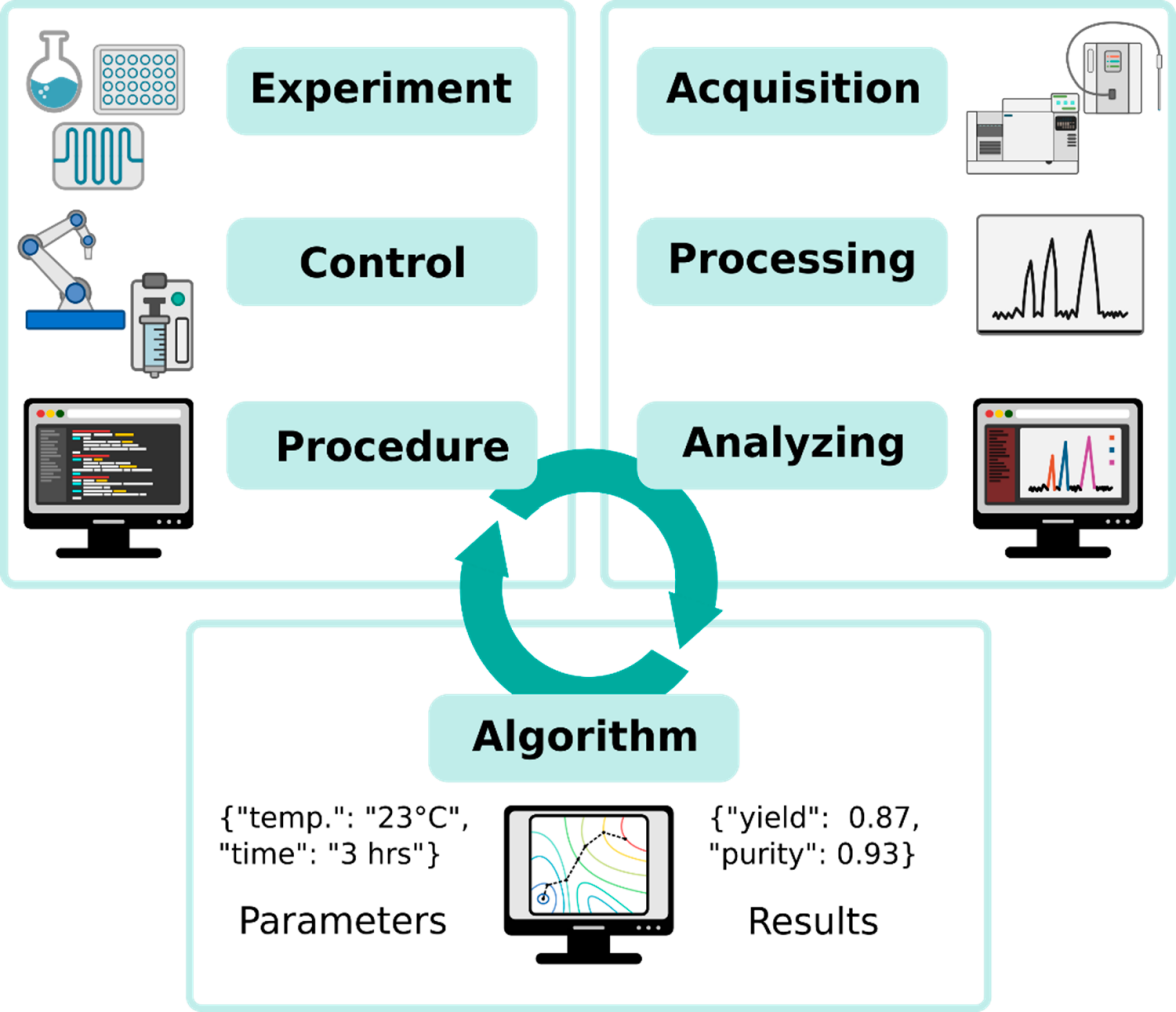
Workflow for the automated chemical reaction optimization: the given procedure is automatically executed on an experimental platform using a corresponding control system; the results of the process are evaluated using an analytical instrument with the resulting data processed and analyzed to yield an outcome that can be understood by an optimization algorithm; the algorithm’s output is used to update the procedure parameters and thus start the next iteration of the process.

Process optimization is among the most tedious and labor-intensive tasks within scientific and engineering disciplines, and chemistry is no exception. Even achieving satisfactory reaction conditions is nontrivial and can take weeks of experimentation for a human researcher.^20,23,129^ Most recent developments in reaction automation have been applied to facilitate optimal parameters discovery, however the vast majority of the published platforms are bespoke systems, capable of performing single reaction optimization in flow.^130^ These platforms, despite demonstrating proof-of-concept results, are typically limited to a specific task, while creating a universal, fully automated framework remains challenging (Figure 8). In 2018, a reconfigurable system was presented with modules to perform flow chemistry, including temperature control and a photoreactor, that can run closed-loop optimization in conjunction with various analytical instruments.^7^ The same year OpenFlowChem—a platform for flow chemistry automation, providing communication protocols for analytical instruments and control systems—was demonstrated to run the process of self-optimization using a PID algorithm, as well as predicting the optimal reaction conditions for a semihydrogenation reaction.^131^ A significant disadvantage of this approach is the usage of commercial software such as LabVIEW for instruments control, and experiment management accompanied by tools for data processing and analysis, most commonly Matlab.^96,132^ While this software suite can create closed-loop experiments with analytical feedback and an optimization algorithm generating new parameters, the setup is not easily transferrable across automation platforms due to proprietary licenses which are not widely accessible.

Recently two new approaches to chemical optimization were reported, Summit^133^ and Olympus,^134^ benchmarking frameworks offer a large set of optimization strategies together with virtual benchmarks, and an experimental planning toolkit. In contrast to the aforementioned software, these frameworks are fully open-sourced and due to their open interface, it is possible to plug in any given algorithm, and this could be universally adapted to any typical automation platform.

### 5.1 Data Processing/Treatment

The foundation of a closed-loop reaction optimization system needs to be the automated processing and analysis of the analytical data in real time.^99^ Any reaction outcome (i.e., product spectrum) should be translated into distinct descriptors (i.e., product yield) for the optimization algorithm to process. Initial processing, for example, noise reduction or baseline correction, could be performed within an automated system by the instrument operating software through either manually written macros or an application programming interface (API). Given that the data format specification is provided by the manufacturer, the resulting output may be further analyzed using third party software which may be included in the automation workflow. The LabVIEW/Matlab suite is a common choice to process the analytical data and utilize the output for the mathematical optimization.^135^ With the necessary drivers provided to control the instruments, it can create a robust environment for the reaction optimization, although only available under a proprietary license. With the emergence of open-source software, several packages were developed to process data from a variety of analytical instruments, including HPLC^136^ and NMR.^137^ Such programs often include the graphical user interface (GUI) and a programming interface for seamless integration into any chemical automation platform. With the source code open to the community, the software can be extended with the novel processing algorithms and accommodate new instruments on the market, if this is permitted by the license agreement for the latter.

Manual product assignment is a common approach to selecting the reaction outcome where the reference sample is analyzed by the human expert, and the respective descriptors are “hard coded” into the experiment workflow. However, manual analysis is not suitable for a fully autonomous systems, and full assignment of data on a novel compound or reaction can be time-consuming for either those discovered experimentally or predicted using a retrosynthetic analysis. Recently, supervised and unsupervised machine learning techniques were proposed to aid with identifying signals of interest from raw spectra or direct assignment of ^13^C and ^1^H spectra to proposed structure;^138−141^ however, these approaches were not applied in context of reaction automation. This is, thus, a very attractive direction in the creation of a fully autonomous system for discovery of new materials and synthetic routes and subsequent optimization within the same system.

### 5.2 Algorithms for Decision Making

An extremely important part of the reaction optimization is minimizing the total number of time-consuming experiments that often involve expensive reagents by maximizing the information learned at each iteration. Design of experiments (DoE) was one of the first techniques to formalize the screening process and is used to build a model that describes the relationship between experimental inputs (e.g., reaction temperature or catalyst loading) and outputs (e.g., yield or product purity).^1,2^ This approach can guide optimization by mapping the optimal conditions or initiate exploration of a search space for more sophisticated algorithms to exploit.

**Figure 9:**
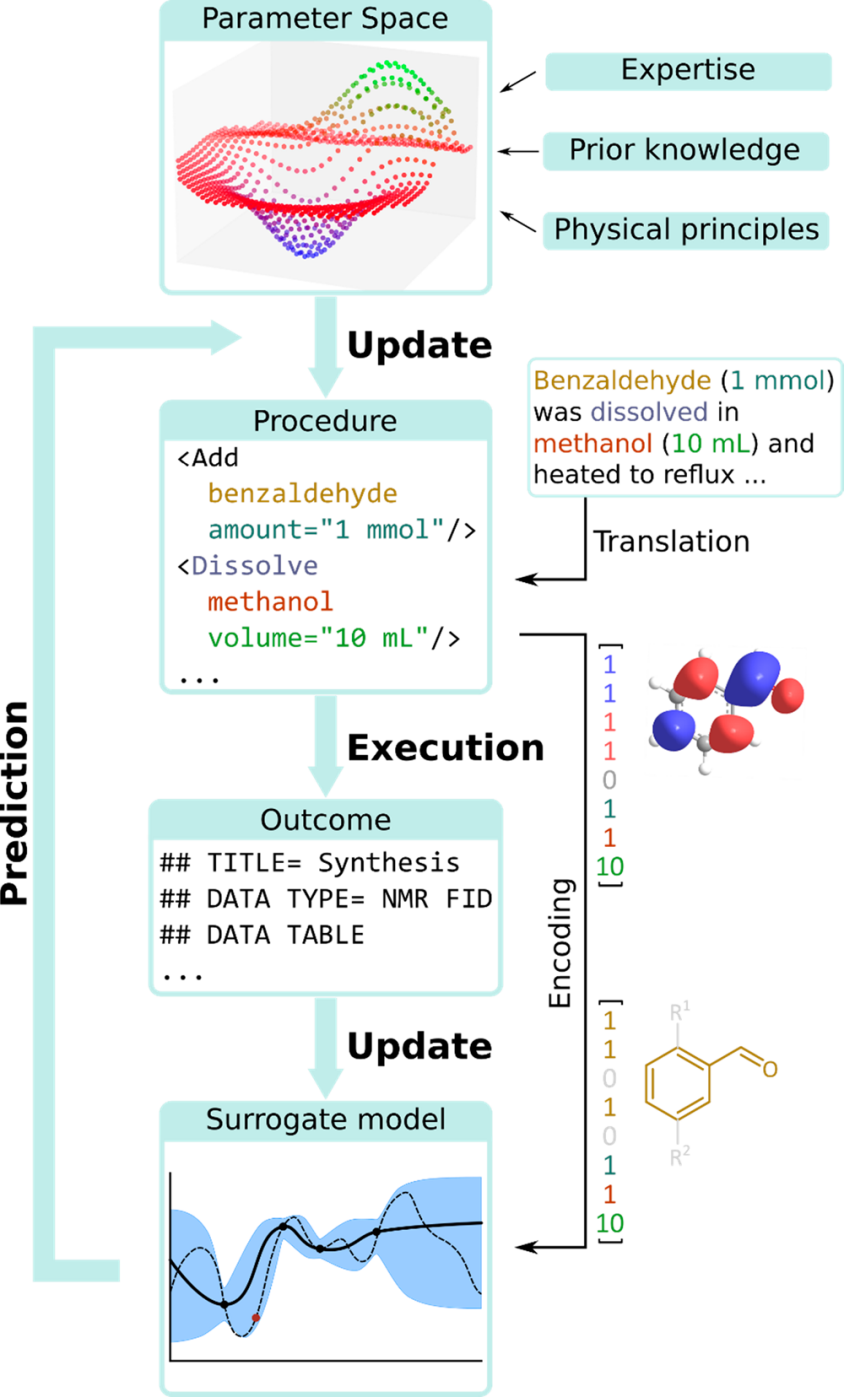
Workflow of the Bayesian reaction optimization: the reaction code, predefined by the parameter space which may be partially informed by prior knowledge and expertise, is executed to result an outcome (illustrated as JCAMP-DX NMR spectrum header); the outcome is then used to update the surrogate model, built using the encoded chemical and conditions data, to predict the next set of reaction conditions.

Traditional algorithms for a function optimization, such as Nelder–Mead Simplex (and modifications thereof) or gradient-based methods, were among the first used in chemical self-optimization tasks.^142^ The significant disadvantage of these local optimizers is their inability to tackle experimental noise which can lead to the premature halting of the optimization far from the true system optimum. Furthermore, the overall growth the complexity of a given process, and an increase in the amount of input parameters, may lead to multiple optima, where such local optimization algorithms are not applicable, despite their robustness and small computation times. The SNOBFIT algorithm was one of the first techniques created to target global optimization of noisy and expensive-to-evaluate functions (i.e., experiments).^143^ Despite the limitation on number of input parameters and their continuous nature, the overall performance and availability made SNOBFIT the go-to method for running self-optimization experiments, and a reference for future single-objective optimizers. More recently, a different category of algorithms has gained increased attention in machine learning community. Bayesian optimization utilizes a surrogate model as an approximation of an experiment or simulation that is updated with more sample data in conjunction with an acquisition function (Figure 9).^144^ These methods were designed for the global optimization of noisy “black box” functions that are expensive to evaluate and have been adapted for use within an experimental environment.^145^ It was also shown that this algorithm can guide an automated flow system to find optimal conditions between multiple competing outcomes.^146^ Discrete and categorical parameters, such as solvent or catalyst choice, represent a significant portion of the input variables for chemical processes and are major challenge in developing optimization algorithms. Previously only available using the DoE approach and the response surface methodology,^147^ such variables can be easily incorporated into Bayesian optimization due to flexibility of surrogate models.^148^ Another advantage of this approach is reduced number of iterations needed to achieve the best outcome. It was reported that optimal conditions can be found after exploring only tiny fraction of parameter space.^5^ It is worth noting, that despite current trends and recent developments, one should not be biased when selecting a strategy to solve the optimization task. Traditional machine learning methods and classical mathematical algorithms for function minimization should also be considered, if they can provide an efficient solution. A more detailed description and general overview of algorithms used in chemical optimization are presented in reviews by Bourne and co-workers,^149^ Houben and Lapkin,^135^ and Cronin et al.^18^

One major challenge for current chemical optimization is incorporating chemical knowledge (e.g., reagent structure, solvent, catalyst nature, etc.) into the overall workflow. Despite significant advances in retrosynthetic planning,^150^ machine learning methods that encode the chemical structure for predictions have not found a wide application in optimization tasks. Most algorithms treat the chemical reaction as a “black-box function”, not only ignoring the physical principles of the reaction, but disregarding data from previously published results or chemical databases in the initial modeling. Previous attempts to incorporate structural knowledge for predicting reaction conditions were based on nearest-neighbor approach, which recommended similar conditions for similar substrates.^151^ The neural network model for encoding structure information has however demonstrated in silico efficiency in predicting suitable reaction conditions when trained on large chemistry databases.^152^ When combined with Bayesian optimization methodology, chemical encoding (achieved using DFT-descriptors) shows excellent performance across several chemical reactions, compared to traditional DoE approach or human expertise, when applied to mixed categorical-continuous parameter domains.^6^ Studies with deep reinforcement learning have shown that a model trained on a specific reaction can also be transferred to a similar or different reaction class to improve the performance and reduce the number of experiments to identify the best conditions.^153^ Overall, this paradigm for the chemical optimization opens a new perspective for predicting the optimal parameters for known reactions but also suggests the conditions for yet unknown reactions, designed using algorithmic retrosynthetic analysis.^154^

## 6 Conclusions

It is clear from the work described in this Perspective that automation is already ubiquitous in the chemical lab environment. From autosamplers to flow reactors, chemists around the world are taking advantage of the labor-saving benefits and increased reproducibility of robotic systems. Thus, the digitization of chemistry should not merely be considered a problem for the future but a challenge of the present. However, there are still strides to be made to reach full automation of chemical optimization or even fully autonomous laboratories, which can discover, optimize the synthesis of and analyze the mode of action of a new lead compound. Comparisons can be drawn with other technological advances, such as cars: when first steam prototypes were built, they were rejected by society as noisy, dangerous and destructive to roadways. With early developments of gasoline engines, cars were still considered to be less practical than common horse wagons, because of regular stalling and high demand in servicing. A significant stream of technological advances (e.g., four-wheel brakes, independent suspension and three-point seat belts), as well as standardization of mass production led to cars that are robust, cheap, and safe enough to dominate the field of private transport. Several decades ago, car owners required basic mechanical skills for everyday maintenance, while nowadays cars can self-diagnose a range of internal faults. With the current rate of development of self-driving cars, in few years one might not even need a license to drive. Chemistry is poised to undergo a similar “great leap forward” by drawing upon the automation and computational advantages described above but there are still several key hurdles to be overcome to transition the remaining areas of manual input to fully autonomous systems (Table 3). Integration and interoperability of hardware is vital, including the presence of standardized hardware interfaces for control and data transfer as well as open access API to empower flexible use of hardware. Another major hurdle for the more elaborate automated workflows is the reliability of current systems, and here we expect real-time monitoring for error detection and correction to play a vital role in guaranteeing the quality of robotically generated reaction data sets. We also urgently need data standards integrated into our publication and output systems, whereby data is open access, acquisition parameters are fully detailed, and the data is in a simple machine-readable format, subject to verification checks and easily searchable using nonproprietary databases. Importantly, this data must include details of automated hardware used for synthesis and the precise action sequence followed in a universally readable format. Missing values and ambiguities (e.g., vigorous stirring versus 1000 rpm) in published data lead to unnecessary barriers when adopting literature procedures to automated platforms. Sharing digital code, such as χDL, is likely to enhance knowledge transfer and scientific collaboration especially when these digital tools are accessible for researchers without programming skills. This can be facilitated by developing web applications and GUIs or interfacing laboratory automation control software with popular, existing workplace tools. Finally, since research is ultimately a human endeavor researchers must have access to training in digital skills and clear directions on how automation tools are used in cutting edge research such that the barrier to adoption of these tools is lowered. Only when the expertise of chemists is combined with the advances allowed by digital tools will the promise of the digital chemistry revolution be fulfilled.

**Table 3:** Challenges and Potential Solutions for the Main Factors in Successful Automation of Optimization of Chemistry

topic	challenge	solution
optimization	algorithm implementations not suited for laboratory use	unified ask-and-tell interface
	chemistry often treated as a black box	big data analysis to gain new insights into reactivity
	high experimental cost, optimization only out of necessity	incorporate prior knowledge for optimizations with lower experimental budgets
execution	transparency and reproducibility	specify device capabilities and limitations
	no plug-and-play automation	establish an open standard
	reliability	control software with error correction
	interoperability	learn the “delta” between different platforms from empirical data
collaboration	slow knowledge transfer	share digital code so that optimized procedures can be directly used in a different lab
	scalability	distributed clients working together via a central server
	programming	no code, GUI, webapps, slack integration
	no platform fits all needs	modular platforms, cross-platform code portability
data management	disconnect between variables and actual actions	record action sequences, map variables
	different research cultures, formats, etc.	human and machine-readable format, FAIR principles
	missing values and procedural ambiguity reduce reproducibility	synthetic chemistry experts needed to bridge the gap

To fully leverage the time saving ability of digitization, in the future chemical processes must be able to be optimized autonomously by integration of closed-loop feedback processes in concert with state-of-the-art algorithms. Given the expensive and complex nature of chemical experiments, algorithms should provide an interface that allows control over the optimization loop and frameworks, such as Summit and Olympus, are expected to prove highly useful for closed-loop systems. When combined these advances will revolutionize scientific collaboration and innovation not only within chemistry but in a wide range of downstream applications such as pharmacy, materials, food technology and energy ultimately bringing the central science into the 21st century.

## Notes

The XDL standard code is freely available from gitlab: https://gitlab.com/croningroup/chemputer/xdl and the documentation is available at www.xdl-standard.com.
